# Strategies for Coping With Stress Used by Nurses in Poland and Belarus During the COVID-19 Pandemic

**DOI:** 10.3389/fpsyt.2022.867148

**Published:** 2022-04-27

**Authors:** Krystyna Kowalczuk, Andrei Shpakou, Justyna M. Hermanowicz, Elzbieta Krajewska-Kułak, Marek Sobolewski

**Affiliations:** ^1^Department of Integrated Medical Care, Medical University of Bialystok, Bialystok, Poland; ^2^Department of Theory of Physical Culture and Sport Medicine, Yanka Kupala State University of Grodno, Grodno, Belarus; ^3^Department of Pharmacodynamics, Medical University of Bialystok, Bialystok, Poland; ^4^Department of Clinical Pharmacy, Medical University of Bialystok, Bialystok, Poland; ^5^Faculty of Management, Rzeszow University of Technology, Rzeszow, Poland

**Keywords:** nurse, wellbeing, stress, coping strategies, pandemic COVID-19

## Abstract

**Introduction:**

Stress is an inseparable element of nurses' work. It is also the cause of wellbeing disorders and the source of various diseases. The wellbeing and health of nurses has a direct impact on the quality of care and health outcomes for patients. An appropriate stress coping strategy can reduce the impact of stress and mitigate its negative consequences. The COVID-19 pandemic, especially in its initial period, was a source of enormous additional stress for nurses. In Poland and Belarus: two neighboring countries with common history and similar culture, the authorities took a completely different approach to fighting the COVID-19 pandemic.

**Aim:**

The purpose of this study was to investigate and compare how nurses in Poland and Belarus cope with stress during the COVID-19 pandemic.

**Materials and Method:**

The cross-sectional study was conducted among 284 nurses working in hospital in Bialystok, Poland (158) and in Grodno, Belarus (126). Mini-Cope inventory - the polish adaptation of Carver's BriefCope was used for measuring coping with stress.

**Results:**

Only 17.5% of Belarusian nurses were tested for the presence of the virus and only 4.8% were infected, while in Poland it was 50.6 and 31.0%, respectively. The most frequent used coping strategies were active strategies (*active coping, planning*) and the least-used were avoidance strategies (*behavioral disengagement, substance use*) in both countries. Polish nurses significantly more often than Belorussian used support-seeking/emotion-oriented strategies, as well as avoidance strategies. No differences were found for active coping strategies between the both groups. Contact with a patient infected with the SARS-CoV2 virus did not influence the choice of stress coping strategies by nurses in both countries. Staying in quarantine or home isolation favored more active coping strategies, especially in the case of Belarusian nurses. Taking a SARS-CoV-2 test did not statistically differentiate the choice of coping strategies in the Belarusian group. In the Polish group, nurses with a positive SARS-CoV-2 test result used both *use of instrumental support* and *use of emotional support* strategies less frequently. SARS-CoV-2 virus infection did not statistically differentiated how stressful situations were handled in Polish group.

**Conclusions:**

Polish and Belorussian nurses used similar strategies to cope with stress in the face of the COVID-19 pandemic. The social and demographic differences between Polish and Belorussian nurses differentiated the choice of coping strategies among the respondents to a greater extent than the completely different approach of the media and authorities to the COVID-19 pandemic in the two countries. The threat of the COVID-19 pandemic does not affect the choice of stress coping strategies by nurses in Poland and Belarus. Being in quarantine or home isolation favored the use of active coping strategies among Belorussian nurses. Polish nurses, on the other hand, were more likely to turn to religion after being quarantined.

## Introduction

Over the last decades, the focus of research has been less and less on the experience of stress, and more on the activity that a person undertakes to cope with stressful events, referred to as coping with stress. The effects of stress are determined more by coping than by the objective properties of the stressor (1–3). Most of the research conducted in this area refers to the transactional view of stress (4), formulated by Lazarus and Folkman, which assumes that stress is a disruption of the relationship (transaction) between the individual and the environment (4, 5).

Assessing a situation in terms of stress entails assessing the possibility of taking action to remove the causes of the stress or mitigate its effects. A person assesses their abilities, competencies, social support, and material resources that may be useful in restoring balance between them and the environment (6). This assessment, called secondary appraisal in Lazarus and Folkman's conception, becomes the starting point for actions directed at changing the stress transaction, which has come to be known as stress coping (7). Coping with stress is a process involving the entirety of an individual's efforts to cope with a given stressful situation. Lazarus and Folkman distinguished two main functions of coping, i.e., instrumental, associated with problem-focused coping styles, and regulatory, associated with emotion-focused coping strategies (7). The former serves to control the stressor in order to reduce or remove its stressful properties, while the latter helps to control the emotional response associated with the stressor.

Referring to the transactional stress theory, Endler and Parker distinguished three styles of coping with stress (8, 9). The first two, task-oriented and emotion-oriented, correspond to the functions identified by Lazarus and Folkman. The third style, avoidance-oriented, serves primarily to reduce the effects of the stressor. It is the most adaptive style and is more effective in dealing with short-term problems than chronic stress situations, such as long-term illness.

An interesting approach to coping with stress, being a combination of coping understood as a style and as a strategy, was presented by Carver et al. (10). The authors, referring to Lazarus' theory (5) and to the model of self-regulated behavior (11), proposed a dozen coping strategies that may reflect both a certain fixed tendency to cope in a certain way (*dispositional coping*) and ways used in a specific stressful situation, referred to as *situational coping*. According to Carver et al., coping results from the characteristics of the individual and the situation, hence it was assumed that the measurement tool should differentiate between *dispositional coping* (a style that is a relatively fixed disposition of an individual) from *situational coping* (an individual's specific coping in a given situation) and measure a variety of avoidance and active, cognitive and behavioral, problem-focused and emotion-focused strategies. This resulted in the construction of the COPE *(The Coping Orientations to Problems Experienced*) questionnaire, consisting of 60 statements comprising 15 coping strategies (10). In 1997, Carver presented an abbreviated version of the BriefCOPE inventory consisting of 28 statements comprising 14 coping strategies (12), the Polish adaptation of which, called MiniCOPE, was used in the present study (13).

The study was conducted among nurses, who, according to studies performed in many countries, constitute one of the most stressed professional groups among professions practiced around the world (14, 15). In a study conducted among Polish nurses, between 73.6 and 95.6% of the respondents stated that the nursing profession is stressful (16, 17), and more than 56.1% of the respondents believed that they are exposed to stress at work every day (18). Factors influencing nurses' perceptions of occupational stress are mainly shift-based work, interpersonal conflicts with other staff members, role conflict, lack of physician support, low autonomy levels, little or no control over work performance, rotation between hospital departments, and aggressive behaviors from coworkers and patients (19). Additional factors that exacerbate stress are contact with seriously ill patients and exposure to death and dying (20, 21).

Nurses' long-term exposure to stress has a negative effect on mental health and also leads to behavioral problems (22). Adverse health behaviors resulting from occupational stress include sleep disturbance, smoking, alcohol and drug abuse, as well as absenteeism from work (23). Long-term exposure to stressors can also lead to fully symptomatic burnout syndrome and depression (24). Furthermore, long-term studies among nurses have documented a strong association between stress and chronic disease (25).

The effectiveness of nurses' coping techniques affects their health and wellbeing. Nurses who experienced high levels of stress, while not actively coping with stress, had the worst health outcomes and the most health risk behaviors compared to other nurses (26, 27).

Each stress reaction is experienced subjectively by an individual depending on, e.g., their psychophysical condition, the type and location of the stress reaction, age, education and knowledge of coping skills. Individual perceptions of stress change with age, working hours, experience, and the overlap of other difficulties, such as problems at home, on the perception of a current or past problem (28).

Undoubtedly, the emergence of the COVID-19 pandemic has been a tremendous source of stress for entire populations and, most importantly, for healthcare workers, including nurses (29). Authorities in different countries have approached the fight against the virus differently. Media coverage on the mortality rate, the severity of illness caused by the virus, the condition of health services, as well as other effects caused by the virus, and the effects of restrictions on civil liberties also varied.

The study was conducted in November 2020 during the so-called second wave of the epidemic in Poland. This wave of infections was actually the first one in Poland, because in the initial period of the pandemic, i.e., between March and May 2020, there were about 300–400 infections a day detected in Poland, and there were 25,000 daily infections in November (30). During that time, Poland was in lockdown. Schools and colleges moved to remote learning, shopping malls and restaurants were closed, limits were placed on people in stores, churches and transportation, gatherings and mass events, and even family celebrations were banned, and state borders outside the Schengen group were closed (31). Popular media focused on reporting the darkest sides of the pandemic and the lockdown. Statistics on the number of infections, deaths, vacant hospital beds and ventilators were cited daily. The media reported on the construction of temporary hospitals and speculated whether the Polish health system would cope with the epidemic.

A second wave of infections was also rising in Belarus at that time. Approximately 1,500 cases of infections were detected daily, a number four times lower than in Poland, given the four times smaller population (32). In November 2020, the Belorussian authorities did not introduce a lockdown, but only recommended keeping social distance and wearing masks. The state media did not make the pandemic a headline in the daily news, but rather tried to show that the pandemic was hardly affecting Belarus, and that the Belorussian healthcare was perfectly prepared (33).

There have been many studies confirming that the COVID-19 pandemic is a greater source of stress for healthcare workers than it is for the general population (34, 35). There are also studies examining how healthcare workers in different countries cope with stress during the COVID-19 pandemic and what stress resilience programs are being implemented (36, 37). This study attempts to fill a gap in the literature on direct comparison, using the same research tool how nurses, as representatives of frontline health care workers, cope with stress in two neighboring countries with a similar culture but a completely different attitudes of their authorities toward COVID-19 pandemic. In addition, the issue of coping with stress by health care workers in Belarus during the COVID-19 pandemic has not been studied so far.

This study attempts to answer the question whether the strategies of coping with stress used by nurses working in culturally similar countries, but with a different approach of the authorities to the COVID-19 pandemic, differ, and whether there is any relationship between the choice of coping strategies and the COVID-19 pandemic elements.

## Materials and Methods

The cross-sectional study was conducted in November 2020, in Białystok, Poland and in Grodno, Belarus. It involved 284 registered nurses working in Białystok University Clinical Hospital and in Grodno Regional Clinical Hospital. Study procedures were approved in Poland by the Bioethical Commission of the Medical University of Bialystok, ref. no APK.002.344.2020 and in Belarus by the management of the State University of Grodno and the Chief Doctor of the Grodno Regional Clinical Hospital. All procedures performed in studies involving human participants were in accordance with the international ethical standards, which includes voluntary participation, informed consent, anonymity, confidentiality, potential for harm, and results communication.

### Study Group Selection

Due to restrictions in access to hospitals in Poland at the time of the study, as well as general difficulties in organizing face-to-face meetings, the study was conducted only at the University Clinical Hospital in Białystok. Also in Grodno, only one hospital was selected - the Grodno Regional Clinical Hospital. Both cities are of similar size (300'000–360'000 inhabitants), have a similar administrative status as provincial capitals, and are academic centers. The selection criterion was employment in the hospital based on a full-time contract. Nurses working part-time and on other basis than an employment contract were excluded.

### Study Protocol

The study was conducted using paper-based questionnaires. The questionnaires were delivered by the researchers to the hospital, and then distributed to the nurses by the departmental nurses. Participation was voluntary. Before the study, each nurse was informed that the research was anonymous, and that they could withdraw from it without stating a reason. They were asked to complete the surveys in their free time within a week and bring back the completed questionnaires in an enclosed sealed envelope. Two hundred questionnaire surveys were distributed in each hospital, out of which 158 correctly completed questionnaires were returned in Poland and 126 in Belarus, with response rates of 79 and 63%, respectively. The reasons why 116 respondents dropped out of the survey are not known. All the demographic and occupational data were obtained from the surveys in the form of respondents' self-reports. No incentives were used to encourage participation in the study.

### Description of the Questionnaire and the Applied Measures

The research instrument for measuring stress coping was the Mini-COPE multidimensional inventory (13), a Polish version of BriefCOPE: an abbreviated version of Carver's COPE inventory (12). Carver designed her multidimensional COPE inventory based on the Lazarus model of stress (5) and the model of behavioral self-regulation proposed by Scheier and Carver (11). The Mini-COPE inventory measures 14 coping responses. These can be grouped into three key stress management strategies: active coping, avoidance coping, and support-seeking/emotion-oriented coping. The active strategy includes the following responses: *active coping, planning and positive reframing*. The avoidance strategy includes *acceptance, humor, self-distraction, denial, substance use and behavioral disengagement*. The support-seeking/emotion-oriented strategy includes *religion, use of emotional support, use of instrumental support, venting and self-blame*. Each response can be scored from 0 (not used at all) to 3 (often used). These measures were standardized by the authors of the questionnaire. The original BriefCOPE inventory and its Polish version of Mini-COPE have been extensively validated, and both have clear scoring guidelines. Cronbach's alpha coefficients for individual scales range from 0.62 (for the *Venting* strategy) to 0.89 (for the *Religion* strategy).

### Statistical Methods

Statistical analysis focused on assessing differences in the occurrence of COVID-19 pandemic-related items in the Polish and Belorussian populations. The Chi-square test of independence was used to assess the significance of differences in this regard.

To compare the significance of differences in the use of coping strategies (Mini-COPE measures) in the two communities, the Mann-Whitney test was used for detailed measures, and the *t-test* was used for the groups of strategies (due to near-normal distributions). The difference in Mini-COPE measures relative to the presence of items related to the COVID-19 pandemic was assessed in an analogous manner.

Using regression analysis, an in-depth examination of the relationship between the occurrence of elements associated with the COVID-19 pandemic and the use of selected stress management strategies was conducted, including control factors such as nurses' age, education, and marital status. Stepwise regression was used to select optimal models that included only statistically significant variables.

STATISTICA v. 13 was used for the calculations. To facilitate the interpretation of results, statistically significant results were denoted according to the following convention: p < 0.05 (^*^), p < 0.01 (^**^), p < 0.001 (^***^).

## Results

### Study Group

The research group consisted of 284 nurses, 158 of whom worked in the hospital in Białystok, and 126 in Grodno. In the Belorussian group, all respondents were women, while in the Polish group there were also 17.1% men. The Belorussian nurses were also older than the Polish nurses. In the Belorussian group, people under 34 years of age constituted 42.0% of the respondents, while in the Polish group they accounted for as much as 67.1%. Nurses over 51 years of age in the Belorussian group comprised 12.2% of the respondents, and only 6.9% in the Polish group. Polish nurses were generally better educated, because as many as 93.4% of the respondents had higher education, and in the Belorussian group it was only 45.2%. Job seniority is closely related to the age of the respondents, which is why as many as 58.2% of the Polish respondents had short job seniority (up to 5 years) compared to 23.0% of the respondents in the Belorussian group.

The Polish nurses were more likely to have had contact with COVID-19 patients, more likely to have been in quarantine, and far more likely to have been infected with SARS-CoV-2 virus. Contact with COVID-19 patients was the only factor occurring with similar frequency, and affected slightly more than half of the nurses in both countries. Detailed data are shown in Table 1.

**Table 1:** Elements of the COVID-19 pandemic among nurses in Poland and Belarus.

**Elements of the COVID-19 pandemic**	**Country**				***p***
	**Belarus**		**Poland**		
	***N***	**%**	***N***	**%**	
Contact with an infected patient	70	55.6	100	63.3	0.1864
Being on home isolation	20	15.9	65	41.1	0.0000^***^
Being on quarantine	10	7.9	46	29.1	0.0000^***^
Taking a SARS-CoV-2 test	22	17.5	80	50.6	0.0000^***^
Having SARS-CoV-2 virus infection	6	4.8	49	31.0	0.0000^***^

*p – test probability value calculated using the Chi-square test of independence*.

*** *p < 0.001*.

### Coping Strategies in Stressful Situations According to Mini-COPE Among Nurses in Poland and Belarus

#### Stress Coping Responses

A comparative analysis of stressful situation management by nurses in Poland and Belarus was conducted. Statistically significant differences occurred for *religion, humor, use of instrumental support, denial, substance use, behavioral disengagement and self-blame*. The largest differences occurred for *religion, substance use, behavioral disengagement and self-blame*. Respondents from Belarus often displayed a sense of humor in stressful situations, whereas the other behaviors were less frequent than in the nurses from Poland. Overall, Polish respondents took more action in stressful situations. Detailed descriptive statistics (mean, median, standard deviations) are shown in Table 2.

**Table 2:** Descriptive statistics for measures of coping strategies among nurses.

**Coping strategies**	**Country**						***p***
	**Belarus (*****N*** **=** **126)**			**Poland (*****N*** **=** **158)**			
	**Mean**	**Median**	**Std. dev**	**Mean**	**Median**	**Std. dev**	
Active coping	2.07	2.0	0.69	2.03	2.0	0.59	0.5580
Planning	1.86	2.0	0.73	1.96	2.0	0.60	0.1960
Positive reframing	1.77	2.0	0.78	1.63	1.5	0.62	0.0621
Acceptance	1.85	2.0	0.74	1.77	2.0	0.61	0.1962
Humor	1.40	1.5	0.85	1.11	1.0	0.83	0.0045^**^
Religion	0.87	0.5	0.90	1.53	1.5	0.89	0.0000^***^
Use of emotional support	1.77	2.0	0.77	1.85	2.0	0.66	0.3440
Use of instrumental support	1.58	1.5	0.73	1.82	2.0	0.63	0.0054^**^
Self-distraction	1.75	2.0	0.69	1.74	2.0	0.82	0.6333
Denial	0.86	1.0	0.65	1.19	1.0	0.76	0.0002^***^
Venting	1.37	1.5	0.64	1.44	1.5	0.78	0.1944
Substance use	0.40	0.0	0.63	0.95	1.0	0.90	0.0000^***^
Behavioral disengagement	0.87	1.0	0.61	1.23	1.0	0.83	0.0001^***^
Self-blame	0.95	1.0	0.68	1.42	1.5	0.78	0.0000^***^

*p – test probability value calculated using Mann-Whitney test*.

** *p < 0.01*,

*** *p < 0.001*.

#### Key Stress Coping Strategies

A parametric test was used for the three main groups of strategies because, by averaging the values of several measures, they had a distribution close to normal. With this in mind, slightly different descriptive statistics were used: mean value with 95% confidence interval and standard deviation shown in Table 3. Polish nurses had significantly higher levels of support-seeking/emotion-oriented strategies, as well as avoidance strategies. No differences were found for the most rational strategies: those involving active behaviors.

**Table 3:** Descriptive statistics for key group stress coping strategies among nurses.

**Key group strategies**	**Country**				***p***
	**Belarus**		**Poland**		
	**Mean (95% c.i.)**	**Std. dev**.	**Mean (95% c.i.)**	**Std. dev**.	
Active	1.90 (1.80–2.00)	0.56	1.87 (1.81–1.94)	0.42	0.6854
Support-seeking/emotion-oriented	1.31 (1.22–1.39)	0.49	1.61 (1.54–1.69)	0.48	0.0000^***^
Avoidance	1.19 (1.12–1.26)	0.41	1.33 (1.24–1.42)	0.57	0.0188^*^

*p – test probability value calculated using t-test for independent samples*.

* *p < 0.05*,

*** *p < 0.001*.

### COVID-19 Pandemic and the Behavior of Nurses From Belarus in Stressful Situations

#### Correlations of COVID-19 Pandemic Items With MiniCOPE Measures

A comparative analysis of the MiniCOPE measure distribution against the occurrence of contact with a SARS-CoV-2 virus infected patient, the need for isolation or quarantine, the need to undergo a virus test, and the occurrence of SARS-CoV-2 virus infection in the respondent was performed.

##### Contact With a Patient Infected With SARS-CoV-2 Virus

The mere fact of contact with a SARS-CoV-2 virus infected patient did not statistically significantly differentiate stress coping.

##### Isolation or Quarantine

Those who underwent home isolation or quarantine had significantly higher levels of *active coping, planning and use of emotional support*. In other cases, no statistically significant relationships were found. Detailed results are shown in Table 4.

**Table 4:** MiniCOPE strategies among nurses in Belarus who have undergone home isolation or quarantine.

**Coping strategies**	**Home isolation or quarantine**				***p***
	**No (*****N*** **=** **102)**		**Yes (*****N*** **=** **24)**		
	**Mean**	**Std. dev**.	**Mean**	**Std. dev**.	
Active coping	2.00	0.71	2.38	0.56	0.0233^*^
Planning	1.75	0.72	2.29	0.59	0.0011^**^
Positive reframing	1.74	0.79	1.92	0.73	0.4163
Acceptance	1.82	0.77	2.00	0.55	0.3553
Humor	1.42	0.88	1.35	0.71	0.6996
Religion	0.87	0.88	0.88	1.00	0.8314
Use of emotional support	1.67	0.77	2.17	0.67	0.0062^**^
Use of instrumental support	1.54	0.75	1.77	0.64	0.1777
Self-distraction	1.73	0.71	1.81	0.62	0.5678
Denial	0.86	0.66	0.85	0.65	0.9140
Venting	1.35	0.67	1.44	0.50	0.6148
Substance use	0.43	0.66	0.25	0.47	0.2333
Behavioral disengagement	0.88	0.65	0.85	0.40	0.9091
Self-blame	0.97	0.72	0.90	0.49	0.8798

*p – test probability value calculated using Mann-Whitney test*.

* *p < 0.05*,

** *p < 0.01*.

##### Taking a SARS-CoV-2 Virus Test

Taking the SARS-CoV-2 virus test did not statistically significantly differentiate stress coping.

##### SARS-CoV-2 Virus Infection

Only six nurses stated that they had been infected with the SARS-CoV-2 virus. With this group being so small, there would have to be really significant differences compared to the other nurses for it to be considered non-random. This result was obtained only for *use of emotional support*, which was stronger among those who had been infected *(p* = 0.0273^*^).

#### Correlations of COVID-19 Pandemic Elements With Key Groups of Stress Coping Strategies

A comparative analysis of the distribution of stress coping strategies was conducted, relative to the occurrence of contact with a SARS-CoV-2 infected patient, the need for isolation or quarantine, the administration of a virus test, and a SARS-CoV-2 infection in the respondent. The only statistically significant difference was in the use of active strategies, which were more often undertaken in stressful situations by nurses who were in isolation or quarantine. Table 5 shows the mean along with the 95% confidence interval for the strategy groups relative to being in quarantine or home isolation. No statistically significant results were obtained for contact with a SARS-CoV-2-infected patient, a viral load test, and the presence of SARS-CoV-2 infection in the respondent.

**Table 5:** Key groups of coping strategies among nurses in Belarus who have undergone home isolation or quarantine.

**Key group strategies**	**Home isolation or quarantine**		***P***
	**No (*N* = 102)**	**Yes (*N* = 24)**	
Active	1.83 (1.72–1.94)	2.19 (1.98–2.40)	0.0036^**^
Support-seeking/ emotion-oriented	1.28 (1.18–1.38)	1.43 (1.27–1.59)	0.1747
Avoidance	1.19 (1.10–1.28)	1.19 (1.08–1.30)	0.9827

*p – test probability value calculated using t-test for independent samples*.

** *p < 0.01*.

#### Correlations of COVID-19 Pandemic Elements With Stress Coping Strategies With Demographic Factors

Demographic factors such as age, having children, and education may also influence the use of stress management strategies. We constructed fourteen regression models in which the dependent variables were consecutive measures of coping with stress according to Mini-COPE, and the independent variables were demographic factors (age, education, having children, and marital status) and COVID-19 pandemic-induced factors (contact with an infected patient, undergoing quarantine or home isolation). Due to low numbers, the fact of detecting infection and undergoing a viral test were not included. Statistically significant features were selected from the aforementioned list using a progressive stepwise regression procedure.

No statistically significant model was found for most measures, suggesting that neither the COVID-19 pandemic elements nor the demographic characteristics included in the analysis differentiate stress behavior in a statistically significant way. The exception is when the respondents were in quarantine or home isolation. These subjects were more likely to use *active coping, planning and use of emotional support* strategies than those who were not quarantined. Within the demographic factors, only education level differentiated *planning* (those with a tertiary education were more likely to choose *planning*) and age (older nurses were more likely to use *religion, denial, venting and self-blame*). Detailed data are shown in Table 6.

**Table 6:** Coping strategies according to MiniCOPE among nurses in Belarus according to the COVID-19 pandemic elements and demographic factors.

**Coping strategy/independent variable**	***B* (95% c.i.)**	***p***	**β**
**Active coping**	***R*^2^** **=** **4.7%** ***F*** **=** **6.1** ***p*** **=** 0.0152^*^		
Home isolation or quarantine (yes vs. no)	0.380 (0.074; 0.685)	0.0152^*^	0.22
**Planning**	***R*^2^** **=** **12.3%** ***F*** **=** **8.6** ***p*** **=** 0.0003^***^		
Tertiary education (no vs. yes)	−0.292 (−0.540; −0.043)	0.0219^*^	−0.20
Home isolation or quarantine (yes vs. no)	0.610 (0.294; 0.925)	0.0002^***^	0.33
**Use of emotional support**	***R*^2^** **=** **6.4%** ***F*** **=** **8.5** ***p*** **=** 0.0042**		
Home isolation or quarantine (yes vs. no)	0.495 (0.159; 0.831)	0.0042**	0.25
**Religion**	***R*^2^** **=** **3.8%** ***F*** **=** **4.9** ***p*** **=** 0.0279^*^		
Age (years)	0.014 (0.002; 0.027)	0.0279^*^	0.20
**Denial**	***R*^2^** **=** **4.1%** ***F*** **=** **5.3** ***p*** **=** 0.0225^*^		
Age (years)	0.011 (0.002; 0.020)	0.0225^*^	0.20
**Venting**	***R*^2^** **=** **4.8%** ***F*** **=** **6.3** ***p*** **=** 0.0133^*^		
Age (years)	0.011 (0.002; 0.020)	0.0133^*^	0.22
**Self-blame**	***R*^2^** **=** **3.2%** ***F*** **=** **4.1** ***p*** **=** **0.0452**		
Age (years)	0.010 (0.000; 0.019)	0.0452^*^	0.18

*R^2^ – coefficient of determination, F – test statistic and p-value for the significance of the whole model, B – regression coefficient with 95% confidence interval, p-value for significance of each regression coefficient, ß – standardized regression coefficient*.

* *p < 0.05*,

*** *p < 0.001*.

#### Correlations of COVID-19 Pandemic Elements With Key Groups of Stress Coping Strategies by Demographic Features

Similarly, the dependence of stress management strategy groups on demographic factors and the COVID-19 pandemic was analyzed using three regression models.

No statistically significant regression model was obtained for support-seeking / emotion-oriented strategies or avoidance strategies. This result confirms the previous findings from univariate analysis. In contrast, there were two factors in the regression model for active strategies: education and isolation or quarantine. Nurses who had a history of quarantine or isolation had, on average, 0.428 points higher values on the measure of using active strategies. Nurses without tertiary education have 0.247 points lower values of the active strategies measure. The obtained regression model (Table 7) allows us to explain the variability of this measure in the studied group in 11.3%.

**Table 7:** Active coping strategies among nurses in Belarus according to the COVID-19 pandemic elements and demographic factors.

**Independent variables**	**Active strategies** ***R***^**2**^ **=** **11.3%** ***F*** **=** **7.8** ***p*** **=** 0.0006^***^		
	***B* (95% c.i.)**	***p***	**β**
Tertiary education (no vs. yes)	−0.247 (−0.440; −0.055)	0.0122^*^	−0.22
Quarantine (or isolation)	0.428 (0.184; 0.672)	0.0007^***^	0.30

*R^2^ – coefficient of determination, F – test statistic and p-value for significance of whole model, B – regression coefficient with 95% c.i., p-value for significance of each regression coefficient, ß – standardized regression coefficient*.

* *p < 0.05*,

*** *p < 0.001*.

### COVID-19 Pandemic and the Behavior of Polish Nurses in Stressful Situations

#### Correlations of the COVID-19 Pandemic Elements With MiniCOPE Measures

A comparative analysis of the MiniCOPE measure distribution against the occurrence of contact with a SARS-CoV-2 virus infected patient, the need for isolation or quarantine, the need to undergo a virus test, and the occurrence of SARS-CoV-2 virus infection in the respondent was performed.

##### Contact With a Patient Infected With SARS-CoV-2

The mere fact of contact with a SARS-CoV-2-infected patient did not statistically significantly differentiate stress coping.

##### Isolation or Quarantine

Those who underwent home isolation or quarantine had significantly higher levels of *substance use and behavioral disengagement*. In other cases, no statistically significant relationships were found. Detailed results are shown in Table 8.

**Table 8:** MiniCope strategies among nurses in Poland who have undergone home isolation or quarantine.

**Coping strategies**	**Home isolation or quarantine**				***p***
	**No (*****N*** **=** **84)**		**Yes (*****N*** **=** **74)**		
	**Mean**	**Std. dev**.	**Mean**	**Std. dev**.	
Active coping	2.01	0.58	2.06	0.61	0.5950
Planning	1.96	0.59	1.96	0.61	0.9536
Positive reframing	1.68	0.64	1.57	0.59	0.3712
Acceptance	1.83	0.62	1.70	0.60	0.1665
Humor	1.06	0.78	1.18	0.88	0.4094
Religion	1.40	0.94	1.68	0.82	0.0625
Use of emotional support	1.85	0.68	1.86	0.63	0.7627
Use of instrumental support	1.86	0.66	1.77	0.61	0.2123
Self-distraction	1.84	0.82	1.64	0.80	0.0733
Denial	1.11	0.75	1.28	0.75	0.1105
Venting	1.39	0.75	1.49	0.81	0.2235
Substance use	0.80	0.92	1.11	0.86	0.0287^*^
Behavioral disengagement	1.08	0.76	1.41	0.89	0.0080^**^
Self-blame	1.34	0.80	1.51	0.75	0.2452

*p – test probability value calculated using Mann-Whitney test*.

* *p < 0.05*,

** *p < 0.01*.

##### Taking a SARS-CoV-2 Virus Test

The nurses who tested positive for SARS-CoV-2 virus had a significantly lower need to seek instrumental support and a slightly lower (difference close to statistically significant) need to seek emotional support.

##### SARS-CoV-2 Virus Infection

The fact of being infected with SARS-CoV-2 virus did not statistically significantly differentiate how stressful situations were handled.

#### Correlations of COVID-19 Pandemic Elements With Key Groups of Stress Coping Strategies

A comparative analysis of the group distributions of stress coping strategies relative to the occurrence of contact with a SARS-CoV-2 infected patient, the need for isolation or quarantine, the administration of a virus test, and the occurrence of SARS-CoV-2 virus infection in the respondents did not show statistically significant results in any case.

#### Correlations of COVID-19 Pandemic Elements With Stress Coping Strategies With Demographic Factors

Fourteen regression models were constructed in which the dependent variables were consecutive measures of coping with stress according to MiniCOPE, and the independent factors were demographic features (age, education, having children, and marital status) and COVID-19 pandemic-induced factors. In addition to “contact with an infected patient” and “being on quarantine or home isolation”, “taking a SARS-CoV-2 test” and “having SARS-CoV-2 virus infection” were included, as there was a proportion of respondents in the Polish group that allowed this. Statistically significant features were selected from the aforementioned list using a progressive stepwise regression procedure.

For the eight stress responses according to MiniCOPE, the frequency of use depends on the factors considered in the analysis. All of the models have a poor fit to the data, but this is normal in psychometric studies, where there are many subjective, personality-related factors that cannot be accounted for in the analysis. Therefore, what is important is not the degree of fit between the model and the data, but which factors affect behavior in stressful situations and how.

Nurses in contact with SARS-CoV-2 virus infected patients were less likely to use *humor* in stressful situations. The frequency of these behaviors was negatively affected by having a tertiary education. *Religion* was a strategy used more frequently by respondents after isolation or quarantine. No statistically significant correlations were found for the other factors related to COVID-19 pandemic and stress response according to MiniCope.

For demographic factors, *behavioral disengagement and self-blame* were more commonly used by older married people, *substance use and denial* were more commonly used by people with higher education, and *self-distraction* by married people. Detailed data are shown in Table 9.

**Table 9:** Coping strategies according to MiniCOPE among nurses in Poland, according to COVID-19 pandemic elements and demographic factors.

**Coping strategy/independent variable**	***B* (95% c.i.)**	***p***	**β**
**Humor**	***R*^2^** **=** **5.8%** ***F*** **=** **4.5** ***p*** **=** 0.0123^*^		
Contact with an infected patient	−0.295 (−0.571; −0.020)	0.0357^*^	−0.17
Tertiary education (no vs. yes)	0.583 (0.057; 1.108)	0.0300^*^	0.18
**Religion**	***R*^2^** **=** **3.4%** ***F*** **=** **5.2** ***p*** **=** 0.0237^*^		
Home isolation or quarantine (yes vs. no)	0.321 (0.044; 0.599)	0.0237^*^	0.18
**Self-distraction**	***R*^2^** **=** **6.5%** ***F*** **=** **10.2** ***p*** **=** 0.0017^**^		
Marital status (single vs. married)	0.462 (0.177; 0.748)	0.0017^**^	0.25
**Denial**	***R*^2^** **=** **7.0%** ***F*** **=** **11.1** ***p*** **=** 0.0011^**^		
Tertiary education (no vs. yes)	0.804 (0.327; 1.281)	0.0011^**^	0.26
**Substance use**	***R*^2^** **=** **3.5%** ***F*** **=** **5.3** ***p*** **=** 0.0222^*^		
Tertiary education (no vs. yes)	0.675 (0.098; 1.252)	0.0222^*^	0.19
**Behavioral disengagement**	***R*^2^** **=** **12.0%** ***F*** **=** **10.0** ***p*** **=** 0.0001^***^		
Age (years)	0.033 (0.018; 0.047)	0.0000^***^	0.44
Marital status (single vs. married)	0.530 (0.181; 0.880)	0.0032^**^	0.29
**Self-blame**	***R*^2^** **=** **5.5%** ***F*** **=** **4.3** ***p*** **=** 0.0158^*^		
Age (years)	0.015 (0.000; 0.029)	0.0483^*^	0.20
Marital status (single vs. married)	0.511 (0.164; 0.858)	0.0042^**^	0.30

*R^2^ – coefficient of determination, F – test statistic and p-value for the significance of the whole model, B – regression coefficient with 95% confidence interval, p-value for the significance of each regression coefficient, ß – standardized regression coefficient*.

* *p < 0.05*,

** *p < 0.01*,

*** *p < 0.001*.

#### Correlations of COVID-19 Pandemic Elements With Key Groups of Stress Coping Strategies by Demographic Factors

Similarly, the dependence of stress management key strategy groups on demographic factors and the COVID-19 pandemic was analyzed using three regression models.

No statistically significant regression model was obtained for support-seeking / emotion-oriented strategies or active strategies. The only model is for the frequency of using *Avoidance strategies* and only one variable, education, was included. Less educated nurses are more likely (the difference, on average, is 0.456 points) to use avoidance strategies in stressful situations. The regression model can explain 3.9% of the variation in this MiniCOPE measure in the study group.

#### Comparison of the Impact of Elements of the COVID-19 Pandemic on Behavior in Stressful Situations Among Nurses From Poland and Belarus

Using regression analysis, the impact of elements of the COVID-19 pandemic on stress behaviors was assessed among nurses from Poland and Belarus. In the regression models, synthetic MiniCOPE measures were entered as dependent variables, and as potential independent factors: country (Poland vs. Belarus), quarantine (or isolation), interaction of the country and quarantine factors, and two factors related to family situation (having children, and marital status) that could potentially influence psychological response to quarantine. Using a stepwise regression procedure (progressive or backward regression), models containing significant factors and allowing to explain the differences in the frequency of use of each coping strategy in the study sample as much as possible were obtained.

A rather complex model (Table 10) that includes the factor country, quarantine, and the interaction between them was obtained for the frequency of active strategies. Analyzing the values of regression coefficients *(B*), it can be concluded that nurses from Poland use active methods of coping with stressful situations less often, and the more frequent use of this strategy type is affected by being in quarantine, but this effect occurs mainly in Belarus. Among Polish nurses, being under quarantine does not substantially change the value of the measure for the frequency of using active strategies.

**Table 10:** Use of active coping strategies to manage stress depending on the country and quarantine (or isolation).

**Independent variables**	**Active strategies** ***R***^**2**^ **=** **4.0%** ***F*** **=** **3.9** ***p*** **=** 0.0094^**^		
	***B* (95% c.i.)**	***P***	**β**
Country (Poland vs. Belarus)	−0.137 (−0.268; −0.005)	0.0414^*^	−0.14
Quarantine (or isolation)	0.164 (0.033; 0.296)	0.0143^*^	0.16
Country (Poland vs. Belarus) × quarantine (or isolation)	−0.202 (−0.333; −0.070)	0.0027^**^	−0.20

*R^2^ – coefficient of determination, B – regression coefficient with 95% c.i., p-value for significance of each regression coefficient, ß – standardize regression coefficient*.

* *p < 0.05*,

** *p < 0.01*.

A simpler model was obtained for the support-seeking/emotional-oriented strategies (Table 11), where only country dependence is shown. Dependencies on country, age, and marital status were obtained for avoidance strategies (Table 12).

**Table 11:** Use of support-seeking and emotion-oriented strategies for coping with stress by country.

**Independent variables**	**Support-seeking and emotion-oriented strategies** ***R***^**2**^ **=** **8.7%** ***F*** **=** **26.5** ***p*** **=** 0.0000^***^		
	***B* (95% c.i.)**	***P***	**β**
Country (Poland vs. Belarus)	0.298 (0.184; 0.413)	0.0000^***^	0.29

*R^2^ – coefficient of determination, B – regression coefficient with 95% c.i., p-value for significance of each regression coefficient, ß – standardize regression coefficient*.

*** *p < 0.001*.

**Table 12:** Use of avoiding coping strategies by country, age, and marital status.

**Independent variables**	**Avoidance strategies** ***R***^**2**^ **=** **4.4%** ***F*** **=** **4.3** ***p*** **=** 0.0058^**^		
	***B* (95% c.i.)**	***p***	**β**
Age (years)	0.007 (0.001; 0.013)	0.0138^*^	0.17
Country (Poland vs. Belarus)	0.145 (0.019; 0.270)	0.0237^*^	0.14
Marital status (single vs. married)	0.143 (0.006; 0.279)	0.0415^*^	0.14

*R^2^ – coefficient of determination, B – regression coefficient with 95% c.i., p-value for significance of each regression coefficient, ß – standardize regression coefficient*.

* *p < 0.05*,

** *p < 0.01*.

## Discussion

The nature of the nursing profession does not allow for a significant reduction in sources of workplace stress, as confirmed by numerous studies (22, 27, 38). It becomes very important to manage stress in such a situation.

The results of our study showed that nurses in both Poland and Belarus used active strategies of coping with stress most often, with the avoidance strategies being used the least often. This is a very reassuring result because it is most desirable to undertake a proactive coping strategy to maintain good mental and physical health. Emotion-focused and avoidance strategies have a clearly negative impact on a person's mental health and wellbeing. This is supported by the results of studies in the USA (39), Australia and New Zealand (40) and Brazil (41).

Comparing both groups, it can be stated that while the frequency of using active strategies is similar, avoidance and support-seeking/emotion-oriented strategies were undertaken by nurses in the Polish group significantly more often than in the Belarus group. We found the most interesting results for the *humor* and *religion* strategies. *Humor* was the only exception among all avoidance and support-seeking/emotion-oriented strategies, which was significantly more frequently used by Belorussian nurses. However, there was the greatest difference in use of religion among all strategies, favoring Polish nurses. An explanation for this result may be that, according to a survey by the Gallup Institute from 2014, Poland is one of the most religious countries in Europe (nearly 89% of the population profess a religion) (42), while in Belarus more than 50% of the population do not follow any religion (43). A surprisingly similar, albeit opposite, comparison was made in a study conducted in July 2020, also during the COVID-19 pandemic, among nurses in Poland and Iran. In this comparison, it was the Iranian nurses who turned to religion significantly more often, while the Polish nurses used *humor* strategies, which is consistent with the very different role of religion in Polish and Iranian societies (44).

Surveys of nurses in different countries have yielded mixed results. Similar to our study, active stress coping strategies, as occurring most frequently were shown by studies conducted before COVID-19 pandemic (40, 45, 46) and during pandemic (47). Avoidance strategies were the least common in studies conducted before COVID-19 pandemic (45, 46, 48, 49) and during pandemic (47) which is also consistent with the results of our study. Different results were obtained in studies conducted before COVID-19 pandemic among nurses in Malaysia (50) and India (51), where support-seeking/emotion-focused strategies were most popular, and in studies carried out in Australia (52), where avoidance strategies were most commonly used by nurses. In studies conducted during COVID-19 pandemic support-seeking/emotion-focused strategies were most popular in Japan (53) and Italy (54), while avoidance strategies were most commonly used in Greece (55).

It is worth noting here, however, that it is not always possible to directly compare the results of our study with those obtained by other researchers globally, because the ways of coping with stress have not been systematized so far, and therefore no universally used classification of coping strategies has emerged. Numerous studies conducted in nursing populations in Poland and around the world have looked at coping-focused strategies. These include taking steps to identify the problem, seeking information, multiple solutions, weighing the benefits and losses, taking action to modify the situation and acquiring new skills (56, 57). Activities such as reluctance to come to work, venting anger on one's co-workers or family, excessive consumption of coffee, tea, alcohol, use of tranquilizers, seeking the company of others, denying facts to distort reality, as well as the use of distancing, avoidance, selective attention, blaming, seeking social support, exercise, and meditation are described as avoidance strategies in other studies (58). The strategies for seeking support and focusing on emotions include activities such as distancing oneself from the problem, avoiding stressful events, and exercising self-control over feelings and behaviors (59).

When it comes to the Polish group, the results obtained are not significantly different from the results of a survey conducted using the same MiniCope tool among nurses from Bialystok hospitals in 2019, i.e., before the COVID-19 pandemic (60).

To further investigate whether the COVID-19 pandemic somehow influenced nurses' coping with stress, we conducted a correlation analysis of events resulting from the COVID-19 pandemic with Mini-Cope measures. We conducted the analysis separately for the group from Belarus and Poland. The groups were significantly different in terms of age and education, and according to previous research, these factors have a significant effect on the selection of coping strategies (60). In the case of the Belorussian group, a statistically significant correlation was obtained only for the factor of “being in home isolation or quarantine”. Those nurses were more likely to undertake active strategies *(planning and active coping)* and to use *instrumental support*. A comparative analysis of the pooled strategy groups also showed the only significant correlation between “being in isolation or quarantine” and a higher frequency of choosing active coping strategies.

Also in the Polish group, the only factor for which we obtained significant correlations was “being in home isolation or quarantine”. In this case, nurses who spent some time in isolation were more likely to turn to *substance use and behavioral disengagement*: the strategies from the avoidance group. A study among nurses in Italy also found no relationship between caring for patients infected with SARS-CoV-2 and those not infected with the choice of stress coping strategies (54).

Demographic factors such as age, education, having children, and marital status also affect the selection of coping strategies (28, 60). Therefore, we performed multivariate analysis for each group using a set of regression models. The results obtained in the Belorussian group showed an interesting correlation, namely that nurses with a history of home isolation or quarantine are oriented toward more active problem solving *(active coping, planning*), but they also expect more emotional support. In the Polish group, on the other hand, those who had been under quarantine are more likely to turn to *religion*—a strategy from the support-seeking/emotional-oriented group—which also includes the *use of emotional support* common in the Belorussian group. These results were obtained with demographic accompanying variables included in the regression models, so these are not apparent relationships. Religious practices were also highlighted as common in other research carried out during pandemic (44, 61).

Finally, we constructed three complex regression models for the combined results from Poland and Belarus. We included the country, having been in quarantine or home isolation, the interaction of the two, and demographic factors in the set of independent variables. Among the factors associated with the COVID-19 pandemic, we selected only quarantine or isolation, because in previous analyses, the other factors did not show significant correlations with coping strategies. We chose synthetic measures of stress behavior as dependent variables.

Nurses from Belarus used active methods of coping in stressful situations more often than Polish nurses. Polish nurses, on the other hand, resorted to support-seeking/emotional-oriented and avoidance strategies more often than Belorussian nurses. Being in quarantine or home isolation was a factor influencing a more frequent use of active strategies, but this effect occurred primarily in Belarus. We obtained correlations of demographic factors only for avoidance strategies, which are more often undertaken by older and married people in both countries. This result is in line with a recent study carried out during the COVID-19 pandemic (62).

The intensity of the perceived stress has an impact on the health of nurses (39, 63). An appropriate stress coping strategy can reduce the impact of stress and mitigate its negative consequences. It is most desirable to follow strategies of active coping with stress and support seeking since they help to maintain good mental and physical health (40, 41). In particular, support-seeking strategies can be a useful approach to dealing with work-related stress in the context of the COVID-19 pandemic. Improving organizational support, creating a positive workplace climate and stimulating social support from colleagues and managers can play an important role in alleviating the perception of work-related stress and should be a goal for both managers and employees who want to alleviate the negative consequences of stress, both in economic and health terms (64, 65). Mental health of nurses has a direct impact on the quality of care and health outcomes of patients (66), which is especially important during the COVID-19 pandemic.

Summarizing the findings, correlations with the choice of coping strategies could be found only in the case of undergoing quarantine or home isolation, a commonly used instrument to counteract the COVID-19 pandemic. It should be emphasized here, however, that being in isolation for several days or more is conducive to reevaluating one's life choices and making decisions about changes (67). Caring for patients infected with SARS-CoV-2, undergoing testing, and even becoming infected did not affect the Polish or Belarussian nurses' coping with stress, despite completely different approaches to the pandemic by the authorities and media in both countries.

The differences in coping strategies between nurses in Poland and Belarus were minor and mainly due to cultural differences, the role of religion in both societies, and demographic differences i.e., age and education. The influence of cultural and demographic differences has also been demonstrated by other researchers (68, 69).

## Conclusions

- Polish and Belorussian nurses used similar strategies to cope with stress in the face of the COVID-19 pandemic.
- The cultural and demographic differences between Polish and Belorussian nurses differentiated the choice of coping strategies among the respondents to a greater extent than the completely different approach of the media and authorities to the COVID-19 pandemic in the two countries.
- The threat of the COVID-19 pandemic does not affect the choice of stress coping strategies by nurses in Poland and Belarus.
- Being in quarantine or home isolation favored the use of active coping strategies among Belorussian nurses. Polish nurses, on the other hand, were more likely to turn to religion after being quarantined.

## Methodological Limitations

The sample used, study design (cross-sectional study precludes identification of causal relationships between the studied variables) and self-reported style questionnaires are significant limitations of the study. The research was conducted only in single cities in Poland and Belarus. The reasons for not taking part in the study by 21% of invited persons in Poland and 37% in Belarus are unknown due to the manner the study was conducted. The lower level of the questionnaire return rate in the Belarusian group, probably due to the low level of democratization of Belarusian society, which limits the willingness of citizens to express their views in writing, could have made the results biased.

## Data Availability Statement

The raw data supporting the conclusions of this article will be made available by the authors, without undue reservation.

## Ethics Statement

The studies involving human participants were reviewed and approved by Bioethics Committee. Medical University of Bialystok, Bialystok, Poland. Written informed consent for participation was not required for this study in accordance with the national legislation and the institutional requirements.

## Author Contributions

KK: concept of the research, design of the article structure, conducting of the research, review of the references, results analysis, and writing the manuscript. AS: concept of the research, conducting of the research, and results analysis. JH: references search and results analysis. EK-K: design of the article structure and review of the manuscript drafts. MS: statistical analysis. All authors contributed to the article and approved the submitted version.

## Conflict of Interest

The authors declare that the research was conducted in the absence of any commercial or financial relationships that could be construed as a potential conflict of interest.

## Publisher's Note

All claims expressed in this article are solely those of the authors and do not necessarily represent those of their affiliated organizations, or those of the publisher, the editors and the reviewers. Any product that may be evaluated in this article, or claim that may be made by its manufacturer, is not guaranteed or endorsed by the publisher.
